# Editorial: Amyloid-Membrane Interactions in Protein Misfolding Disorders: From Basic Mechanisms to Therapy

**DOI:** 10.3389/fcell.2022.870791

**Published:** 2022-02-25

**Authors:** Neville Vassallo, Céline Galvagnion, Eva Y. Chi

**Affiliations:** ^1^ Department of Physiology and Biochemistry, Faculty of Medicine and Surgery, University of Malta, Msida, Malta; ^2^ Centre for Molecular Medicine and Biobanking, University of Malta, Msida, Malta; ^3^ Department of Drug Design and Pharmacology, University of Copenhagen, Copenhagen, Denmark; ^4^ Department of Chemical and Biological Engineering, University of New Mexico, Albuquerque, NM, United States

**Keywords:** protein aggregation, lipid membrane, amyloid-β, tau, α-synuclein, hIAPP, SOD1, aggregation inhibitors

It is now well established that the aberrant conversion of peptides or proteins from their native functional states into toxic amyloid entities underlies the pathogenesis of a wide range of debilitating human diseases, collectively known as protein misfolding disorders (PMDs). To date, more than 50 amyloidogenic proteins or peptides have been shown to cause PMDs; these include amyloid-β (Aβ) and tau in Alzheimer’s disease, α-synuclein (α-syn) in Parkinson’s disease, superoxide dismutase 1 (SOD1) in Amyotrophic Lateral Sclerosis, and the human islet amyloid polypeptide (hIAPP) in type-2 diabetes mellitus. Accumulating evidence indicates that one of the main pathogenic factors in PMDs involves a direct interaction of amyloidogenic proteins with biomembranes. However, the interplay between the two is highly complex, and overall cytotoxicity depends upon a combination of the physico-chemical properties of the misfolded proteins (e.g., size and surface hydrophobicity) on the one hand, with those of the lipid bilayer (e.g., phospholipid composition, cholesterol and ganglioside content) on the other. Exploring amyloid-membrane interactions at a fundamental level will help inform the development of novel therapeutics for PMDs. In this Research Topic, we have published a collection of five articles dedicated to amyloid-membrane interactions in PMDs, of which three are reviews and two original research articles.

Peptide aggregation occurs along a misfolding continuum, starting from an intrinsically disordered monomeric state, through a range of metastable oligomeric entities, and ending in highly-organised fibrils featuring a repeated cross-β-structure. A consensus is building that the peptide conformations most relevant to toxicity are most likely represented not by the end-stage fibrils, but by the earlier oligomeric intermediates. Thus, Gonzalez-Garcia et al. extensively review recent progress in elucidating mechanisms involved in the interaction between toxic misfolded protein oligomers and biological membranes. Aided by excellent figures, they provide an overview of how oligomers of diverse proteins, such as α-syn, tau, Aβ42, SOD1 and the *Eschericia coli* HypF-N protein, share common mechanisms for disrupting cellular and mitochondrial membranes. These include membrane thinning by partial insertion into the bilayer, formation of transmembrane pore-like structures, and lipid extraction through a detergent-like action. The various mechanisms converge on increased intracellular calcium levels and reactive oxygen species generation, ultimately triggering cellular apoptosis. Oxidative stress serves as a positive-feedback driver of the pathogenic process by causing lipid peroxidation, which in turn instigates further protein aggregation. Gonzalez-Garcia et al. point out that promiscuous misfolded protein oligomers also demonstrate affinity to membrane proteins, for example metabotropic and ionotropic glutamate receptors at synapses, causing an excessive rise in intracellular calcium and excitotoxicity. With regards to mitochondrial proteins, the voltage-dependent anion channel (VDAC) is often a target - aggregates of α-syn, mutant SOD1 and mutant huntingtin all cause VDAC dysfunction and finally activate mitochondrial apoptosis.

The two-dimensional surface of a membrane may powerfully influence the aberrant folding of structurally disordered proteins. Focusing on tau, Sallaberry et al. examine the role of biological membranes in catalyzing tau nucleation and aggregation. Anionic lipid surfaces drive the electrostatic interaction with R2 and R3 microtubule-binding domains of tau, whilst bound tau assumes a compact, β-sheet rich structure. Tau fibrils and aggregation intermediates in turn disrupt lipid packing and compromise cell membrane integrity. Sallaberry et al. also discuss how tau might exit the cell by translocation of small tau oligomers across the lipid membrane, or by exocytosis inside lipid vesicles that bud from the plasma membrane (exosomes). Release of stable tau/lipid aggregates into the extracellular milieu is linked to the spatiotemporal transmission of tau pathology during disease progression, a highly topical subject comprehensively reviewed by Bok et al.

Exosomes, rich in ganglioside lipids, also play a key role in the neuropathological spread of rogue α-syn in Parkinson’s disease. In a research article contributed to this Research Topic, Gaspar et al. use cryo electron microscopy (cryo-EM) to follow the process of co-assembly of α-syn in the presence of ganglioside (GM1)-containing lipid vesicles. Intriguingly, they observed a dynamic role of α-syn in its co-aggregation with lipids, with selective uptake of anionic GM1 into the fibrillar aggregates. In turn, the presence of GM1 in the lipid vesicles influenced the morphology of the end-stage α-syn fibrils. These results have important implications for our understanding of the process of lipid-protein co-assembly in amyloid formation, and demonstrate that the lipid membrane is a dynamic, rather than inert, entity.

An accurate understanding of the aggregation pathway of misfolded proteins allows for the rational design of drug-like organic molecules or peptides that block the self-assembly process. Such as strategy was adopted by Lesma et al. with regards to the highly aggregation-prone hIAPP, involved in type-2 diabetes mellitus. Lesma et al. carried out an in-depth structure-activity relationship (SAR) study of the inhibitory behaviour of flexible, small acyclic β-hairpins. These semi-rigid peptides are based on a piperidine-pyrrolidine β-turn inducer, with specific small peptide arms derived from the hIAPP amyloidogenic sequence. Using an array of powerful biophysical techniques, the authors demonstrate potent selective inhibitory activity of hIAPP oligomerization and fibrillization, as well as protection of lipid vesicles from hIAPP-induced membrane disruption. Altogether, this is a proof-of-concept study validating the inclusion of β-hairpin peptide mimics in the armamentarium of compounds able to inhibit amyloidogenic peptide aggregation.

In conclusion, the publications in this Research Topic provide a taster of the complexity of the molecular mechanisms at play between biological membranes and diverse amyloid proteins, reinforcing the view that such mechanisms represent a unifying theme in PMDs. Furthermore, the amyloid community has to increase its efforts to face the considerable challenge of urgently needed therapeutic strategies for PMDs, by tackling these diseases at their root cause.

